# Dr. Lubbock, on the Publicity of Mayow's Doctrines

**Published:** 1799-07

**Authors:** R. Lubbock


### *To the Editors of the Medical and Physical Journal.*

GENTLEMEN,

GENTLEMEN,

AS your Journal embraces every object connected with medical ſcience, I hope the following obſervations, though only pertaining to the hiſtory of medical literature, will find a place in your next Number.

I am, Gentlemen, your obedient ſervant,

R. LUBBOCK.

No perſon can feel a more lively ſenſe of the value of MAYOW's writings than myſelf; and with that feeling I was induced, in a former Number of your your Review, to add to the few names quoted by Dr. Yeats, many others of great refpectability, proving that, foon after the publication of Mayow's writings, they ftrongly arrefted the attention of the learned in different parts of Europe, and met with as general a diffufion, as ever happened to any work marked with equal originality.

Dr. Yeats has admitted, that Mayow's writings, in a few years after their publication, were quoted by many authors; but his zeal for the rights of Mayow, which is, in many refpects, very meritorious, has led him into fome erroneous conclufions; and more particularly when he infers, that during Mayow's life-time, his doctrine was mentioned by no author; when he indirectly accufes Lower and Thruston of want of candour, in not quoting him; and when he fays, that the authors quoting him, after his death, have many of them miftaken his opinions, by not making a diftinction between fire air particles, and nitre, properly fo called.

It is with much pleafure that I now inform Dr. Yeats, that Mayow was gratified with obferving the progrefs of his difcoveries in Europe, during his life-time; for I find upon a more minute inquiry, that the differtation on refpiration, entitled "Abftrufum refpirationis humanæ negotium, &c." publifhed under the aufpices of Etmuller, and which I formerly quoted from the edition of his works, printed after his death, 1697, was firft publifhed at Leipzig, in quarto, 1676, which period was two years after the appearance of the complete edition of Mayow's works. Oxon. 1674; and three years before his death, 1679.—I quote this differtation with the greater pleafure, as I have met with no work, containing fo full an examination of Mayow's opinions on refpiration, as well as of the opinions of Bartholin, Malpighi, Swammerdam, Thrufton, and others; and of the truth of which I am of opinion every medical reader will be convinced by its perufal: and, as Etmuller filled a profeffional chair at Leipfig, it is more than probable, that in his lectures he detailed and examined the opinions of Mayow on different fubjects, with thofe of other authors.

I might here repeat Mundy's name, who, I have no doubt, mentions Mayow, in his works printed at Oxford, 1680, a year after Mayow's death; and for whofe authority Dr. Yeats would have felt more refpect, had he, with more leifure, examined his chapter on contagion, as well as fome other parts of his work.

Dr. Yeats having faid, in a note to his work, although I believe erroneoufly, that Lower's treatife upon the heart was publifhed many years before Mayow wrote, it is difficult for me to conceive on what ground he cenfures Lower for not citing him. And believing, as I do, that Lower's work appeared appeared for the firſt time, 1699, the year after the publication of the firſt edition of Mayow's Treatiſe on Reſpiration, 1668, and granting Lower's work ſubſequent to Mayow's, by a year, and when the latter was only known, by his treatiſe upon reſpiration and upon the rickets,—I ſee not why the duty of citation ſhould attach to Lower, his experiments and inquiries muſt have been carried on and finiſhed without the knowledge of Mayow's proceedings, and the ſending them into the world, ſupported only by their intrinſic merits, was the act of a mind the moſt ingenious, yet feeling the excluſive right to its own independent labours and exertions.

How far Dr. Yeats is warranted in complaining of Thruſton, for not mentioning the name of Mayow, in his Diatribe, will appear from his own ſtatement, It is admitted by Dr. Yeats, that Thruſton's work was firſt made public, in 1664, as a theſis, before a crouded audience, at Cambridge, and that his principles were generally known by the learned either by converſation, or by a manuſcript copy; for, as Dr. Yeats has obſerved, he expreſſes his obligations to Hook and Lower, for confirming his doctrine by their experiments, and at the ſame time, complains of others, who had publicly attacked it, without mentioning his name. To prove alſo how generally Thruſton's opinions were known, before his work was in print, it may be obſerved, that NEEDHAM wrote a Letter of Congratulation to Thruſton, upon finding that his 'Diatribe' was about to be printed; and alſo that Sir GEORGE ENT, the firſt Phyſiologiſt of the age, wrote his Animadverſions upon Thruſton's work, and had them publiſhed with the firſt edition of it, 1670.—It appears from this ſtatement, that Thruſton's opinions, although not in print, were very generally known among the learned, and of courſe Mayow could be no ſtranger to them, and as they were made public in 1664, four years before Mayow printed his 'Tracts on Reſpiration and the Rickets', it is natural to conclude from theſe facts, in oppoſition to Dr. Yeats, that the duty of citation attached to Mayow; and that inſtead of Thruſton quoting Mayow, Mayow ſhould have mentioned Thruſton. This inference ſeems to ſpring neceſſarily from the above facts; and in his 'Epiſtle to the Reader', Thruſton ſeems to refer to ſome perſons, and perhaps to Mayow, who had anticipated, if not borrowed, his opinions; for in ſpeaking of his work, he ſays, "amiſit illa quidem fortaſſis aliquam novitatis gratiam, quâ ſe olim ſuis auditoribus, aliiſque commendaverit, quainque interim aliorum ſcripta, vel merita fur vel ambierunt."

Neither can I ſubſcribe to the opinion of Dr. Yeats, that CONNOR and TABOR, by the terms *nitrum aëreum*, and *nitrum aëris*, have not made the diſtinction between fire air particles, and nitre as ſuch; by theſe terms, they mean only a principle that air poſſeſſes in common with nitre; and

Mayow

Mayow himſelf makes uſe of the terms *nitrum aereum, particula nitro aerea, & ſpiritus nitro aereus*, indifferently, and as ſynonyms, and upon the ſame ground, according to the modern chemical nomenclature, when oxygen is ſaid to be the principle of acidity, and common to the nitrous and other acids, and to the air, it might be argued, that its nature is not underſtood, the air exhibiting no ſenſible traces of acidity.

NORWICH, *June* 12, 1799.

